# A missense single nucleotide polymorphism in the ALDH2 gene, rs671, is associated with hip fracture

**DOI:** 10.1038/s41598-017-00503-2

**Published:** 2017-03-27

**Authors:** Kenichiro Takeshima, Yuji Nishiwaki, Yasunori Suda, Yasuo Niki, Yuiko Sato, Tami Kobayashi, Kana Miyamoto, Hisaya Uchida, Wataru Inokuchi, Takashi Tsuji, Atsushi Funayama, Masaya Nakamura, Morio Matsumoto, Yoshiaki Toyama, Takeshi Miyamoto

**Affiliations:** 1 Department of Orthopedic Surgery, Keio University School of Medicine, 35 Shinano-machi, Shinjuku-ku, Tokyo 160-8582 Japan; 2 Department of Orthopedic Surgery, Eiju General Hospital, 2-23-16 Higashiueno, Taito-ku, Tokyo 110-8645 Japan; 3 Department of Environmental and Occupational Health, School of Medicine, Toho University, 5-21-16 Omori-nishi, Ota-ku, Tokyo 143-8540 Japan; 4 Department of Orthopedic Surgery, International University of Health and Welfare, Mita Hospital, 1-4-3 Mita, Minato-ku, Tokyo 108-8329 Japan; 5 Department of Advanced Therapy for Musculoskeletal Disorders, Keio University School of Medicine, 35 Shinano-machi, Shinjuku-ku, Tokyo 160-8582 Japan; 6 Department of Musculoskeletal Reconstruction and Regeneration Surgery, Keio University School of Medicine, 35 Shinano-machi, Shinjuku-ku, Tokyo 160-8582 Japan; 7 Department of Orthopedic Surgery, Hiratsuka City Hospital, 1-19-1 Minamihara, Hiratsuka city, Kanagawa 254-0065 Japan; 8 Department of Orthopedic Surgery, Nerima Sogo Hospital, 1-24-1 Asahigaoka, Nerima-ku, Tokyo 176-8530 Japan; 9 Department of Orthopedic Surgery, Fujita Health University, 1-98 Kutsukake-cho, Toyoake city, Aichi 470-1192 Japan

## Abstract

Hip fracture is the most severe bone fragility fracture among osteoporotic injuries. Family history is a known risk factor for fracture and now included among criteria for osteoporosis diagnosis and treatment; however, genetic factors underlying family history favoring fracture remain to be elucidated. Here we demonstrate that a missense SNP in the *ALDH2* gene, *rs671* (ALDH2*2), is significantly associated with hip fracture (odds ratio = 2.48, 95% confidence interval: 1.20–5.10, *p* = 0.021). The *rs671* SNP was also significantly associated with osteoporosis development (odds ratio = 2.04, 95% confidence interval: 1.07–3.88, *p* = 0.040). For analysis we enrolled 92 hip fracture patients plus 48 control subjects without bone fragility fractures with higher than −2.5 SD bone mineral density. We also recruited 156 osteoporosis patients diagnosed as below −2.5 SD in terms of bone mineral density but without hip fracture. Association of *rs671* with hip fracture and osteoporosis was significant even after adjustment for age and body mass index. Our results provide new insight into the pathogenesis of hip fracture.

## Introduction

Osteoporosis is a disease characterized by elevated risk of bone fragility fracture owing to reduced bone strength due to reduced bone mass, reduced bone quality or both^1, 2^. Aging is a great risk factor for osteoporosis development, and thus the number of osteoporosis patients is continuously increasing as the number of elderly people increases in our aging society^3^. In osteoporotic bone fragility fractures, risk factors other than aging include low bone mineral density, estrogen or androgen deficiency, metabolic or inflammatory disease, prolonged steroid use or reported history of parents’ hip fracture(s). Some of these are now utilized as diagnostic criteria to prescribe drugs to prevent bone fragility fractures^4–7^. Although not currently included among criteria for intervention to prevent bone fragility fractures, current smoking, intake of a large amount of alcohol, mutations or single nucleotide polymorphisms (SNPs) in genes that encode the vitamin D receptor (VDR), estrogen receptor (ER), or low-density lipoprotein receptor-related protein 5 (LRP5), and low body weight/low body mass index (BMI) are considered risks for osteoporosis or development of bone fragility fractures^8–11^. In fact, several of these activities are included in calculations of individual risk of fracture within ten years in the fracture risk assessment tool (FRAX^TM^)^12^. Presently, the FRAX^TM^ score is included in criteria for drug interventions to prevent bone fragility fractures^13, 14^. However, specific gene mutations or SNPs associated with hip fracture are not included in these criteria.

Aldehyde dehydrogenase 2 (ALDH2) is a member of a family of enzymes that metabolize alcohol; ALDH2 catalyzes conversion of acetaldehyde to acetic acid^15, 16^. Several *ALDH2* gene SNPs have been identified: among them, *rs671* is a missense mutation, and the resultant mutant ALDH2*2 protein acts in a loss of function or dominant negative fashion^17–19^. Thus, the ALDH2*2 enzymatic activity is severely altered even in heterozygous individuals carrying *rs671* and is reportedly associated with conditions such as alcohol flush syndrome^20, 21^. The frequency of individuals carrying *rs671* differs among human races and is highest in eastern Asia^22^. Serum acetaldehyde levels are relatively and significantly high an ALDH2*2 mouse model compared with control mice even under non-alcohol drinking conditions^23^, suggesting that ALDH2 functions in pathways other than alcohol metabolism. Indeed, *rs671* is associated with diseases as varied as Alzheimer’s disease, esophageal cancer, osteoporosis, cardiovascular disease, gout and Parkinson’s disease^24–30^. In contrast, ALDH2*2 model mice reportedly show stress resistance in response to cardiac ischemia/reperfusion injury^31^. Aldh2-deficient mice exhibit normal bone mass but significantly reduced bone mineral density following alcohol consumption^32^ and increased cortical bone mineral density in the absence of drinking alcohol^33^. We previously reported that ALDH2*2 model mice exhibit significantly reduced bone mass relative to controls without alcohol consumption, although Aldh2 knockout mice show normal bone mass^23^. These results imply that the presence of ALDH2*2 is more potent in reducing bone mass than is ALDH2 deletion.

Here, we investigated *rs671* association with hip fractures. Informed consent was provided by all subjects in the study, and 92 and 48 subjects were enrolled as hip fracture and control subjects, respectively. *rs671* frequency in each group was assessed by direct sequencing. We report that *rs671* is significantly associated with hip fractures even after adjustment for age and body mass index (BMI). The odds ratio for hip fracture was 2.33 (95% confidence interval: 1.02–5.33) based on *rs671*. Our study suggests that *ALDH2 rs671* is a possible risk factor for hip fracture.

## Results

### Research subject characteristics

**Figure 1:**
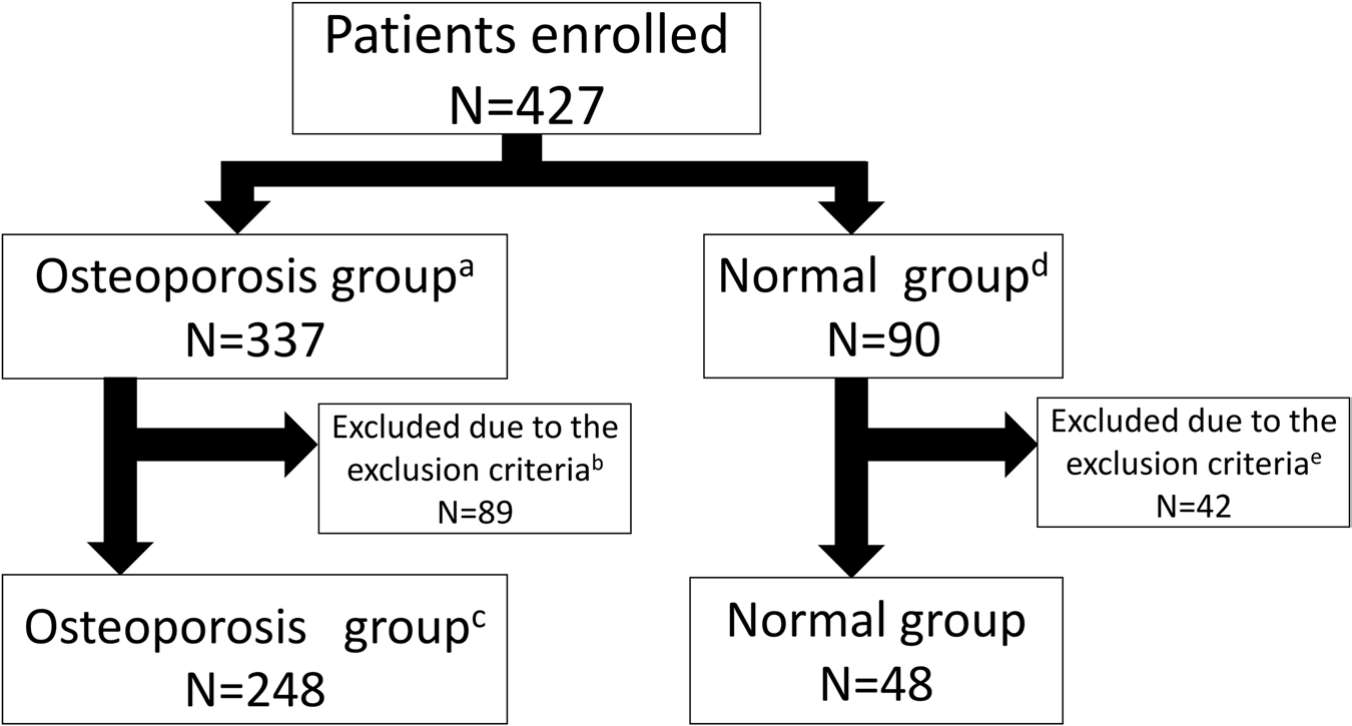
Flow chart of study subjects. Informed consent was obtained from 427 subjects, and 337 and 90 were diagnosed as osteoporosis and normal (non-osteoporosis) based on diagnostic criteria of either bone mineral density lower than a −2.5 SD T-score or current hip fracture history. 89 and 42 subjects were excluded from osteoporosis and normal groups, respectively, due to incomplete datasets or a history of rheumatoid arthritis (RA), diabetic myelitis (DM) cancer, or corticosteroid use. Subjects taking osteoporosis drugs were excluded from the normal group. (**a**) The Osteoporosis group was defined by either lower than a −2.5 SD T-score of BMD or hip fracture. No subjects had previous hip fracture history. (**b**) Cases (89) with history of or under treatment for conditions cited above were excluded. (**c**) Group includes 92 subjects diagnosed as osteoporosis with hip fracture. (**d**) Defined by a T-score greater than −2.5 SD without fragility fracture (includes patients with osteopenia). (**e**) Cases (42) excluded based on criteria cited above.

We enrolled 427 postmenopausal women, who visited or were transported to hospitals and had undergone dual x-ray absorptiometry (DXA) or X-ray examination, for the study. Informed consent was obtained from all. Among them, 337, who were diagnosed as hip fracture or under −2.5 SD BMD, were classified as the “osteoporosis group”, and 90, who did not satisfy osteoporosis criteria, were chosen to represent the “normal group” (Fig. 1). Subjects with a history of or who had been treated for conditions affecting osteoporosis development, such as rheumatoid arthritis or diabetes mellitus, were excluded. In the normal group, subjects who had received osteoporosis medication and had no reliable past BMD data were also excluded. The remaining 248 and 48 subjects served as the osteoporosis and normal groups, respectively. In the former, 92 were hip fracture patients and 156 exhibited low BMD under −2.5 SD and were diagnosed as osteoporosis patients. Significant differences in age, height, body weight and BMI were observed between osteoporosis versus normal groups and between hip fracture versus normal groups (Tables 1 and 2).

**Table 1:** Characteristics of patients with hip fracture group (a subgroup of the osteoporosis group) and control normal patients.

	Hip fracture group (n = 92)	Normal group (n = 48)	p
Age (years)	82.7 ± 11.2 (44–101)	72.8 ± 7.01 (53–87)	<0.001
Height (cm)	149 ± 7.04 (129–170)	153 ± 6.10 (138–165)	0.015
Body weight (BW) (kg)	45.4 ± 8.85 (30–67)	51.8 ± 7.49 (38–69)	<0.001
BMI	20.3 ± 3.61 (13.9–28.4)	22.2 ± 3.00 (17.5–29.1)	0.002

**Table 2:** Characteristics of patients in osteoporosis and normal groups.

	Osteoporosis group (n = 248)	Normal group (n = 48)	p
Age (years)	77.4 ± 10.1 (44–101)	72.8 ± 7.01 (53–87)	<0.001
Height (cm)	150 ± 6.78 (129–170)	153 ± 6.10 (138–165)	0.011
Body weight (BW) (kg)	46.0 ± 7.86 (29–72)	51.8 ± 7.49 (38–69)	<0.001
BMI	20.4 ± 3.32 (13.9–30.0)	22.2 ± 3.00 (17.5–29.1)	<0.001

### Hip fracture subjects carry *rs671* at higher frequency than do normal subjects

**Figure 2:**
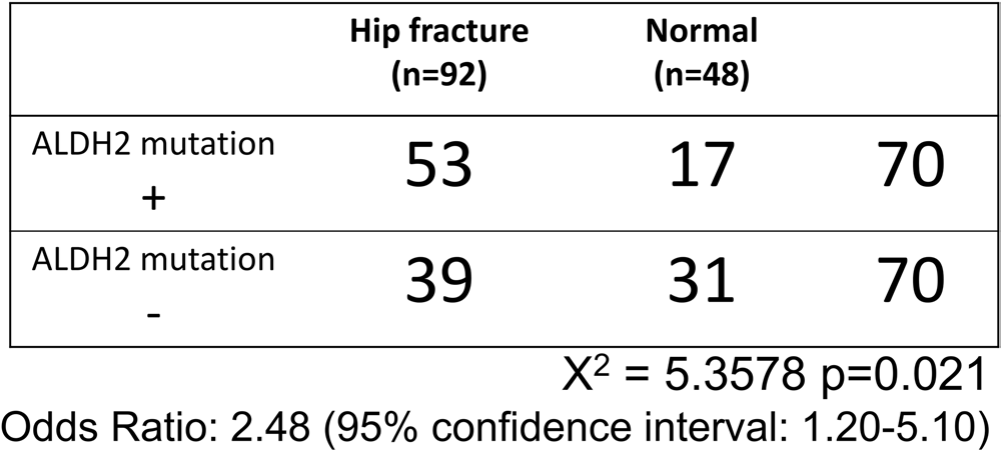
*ALDH2 rs671* mutation in hip fracture and normal groups. The presence of *rs671* in research subjects was analyzed by direct sequencing. Subjects heterozygous or homozygous for the mutation are designated (+) and subjects with the wild-type *ALDH2* gene are designated (−). Odds ratios and 95% confidence intervals for *rs671* in the hip fracture group were determined, and a Chi-square test was performed.

Next we undertook direct sequence analysis to assess the presence of *ALDH2 rs671* in study subjects. Subjects homozygous and heterozygous for *rs671* were classified as ALDH2 mutation (+), and those with the normal gene were ALDH2 mutation (−). Of hip fracture subjects, 57.6% were ALDH2 mutation (+), and the odds ratio was 2.48, with a 95% confidence interval of 1.20–5.10 between hip fracture and normal groups (Fig. 2). Chi-square tests demonstrated that hip fracture was significantly more frequent in the ALDH2 mutation (+) than the (−) group (Fig. 2).

### Osteoporosis subjects carry *rs671* at higher frequency than do normal subjects

**Figure 3:**
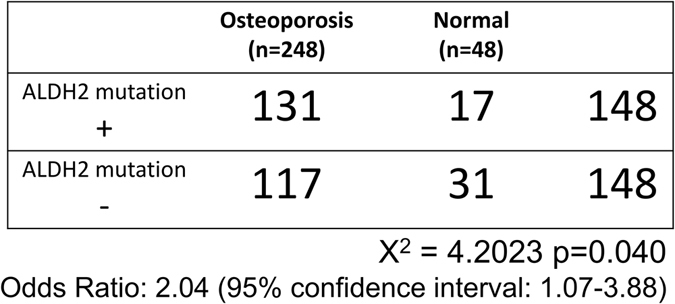
*ALDH2 rs671* mutation in osteoporosis and normal groups. The presence of *rs671* in research subjects was analyzed by direct sequencing. Subjects heterozygous or homozygous for the mutation are designated (+) and subjects with the wild-type *ALDH2* gene are designated (−). Odds ratios and 95% confidence intervals for *rs671* in the hip fracture group were determined, and a Chi-square test was performed.

Next, we analyzed *rs671* frequency in osteoporosis and normal subjects and observed ALDH2 mutation (+) frequencies of 52.8 and 35.4%, in osteoporosis and normal groups, respectively (Fig. 3), with an odds ratio of 2.04 and a 95% confidence interval of 1.07–3.88 between osteoporosis and normal groups (Fig. 3). Chi-square tests showed osteoporosis was significantly more frequent in the ALDH2 mutation (+) than in the (−) group (Fig. 3).

### *rs671* is associated with hip fracture after adjustment for age and BMI

**Figure 4:**
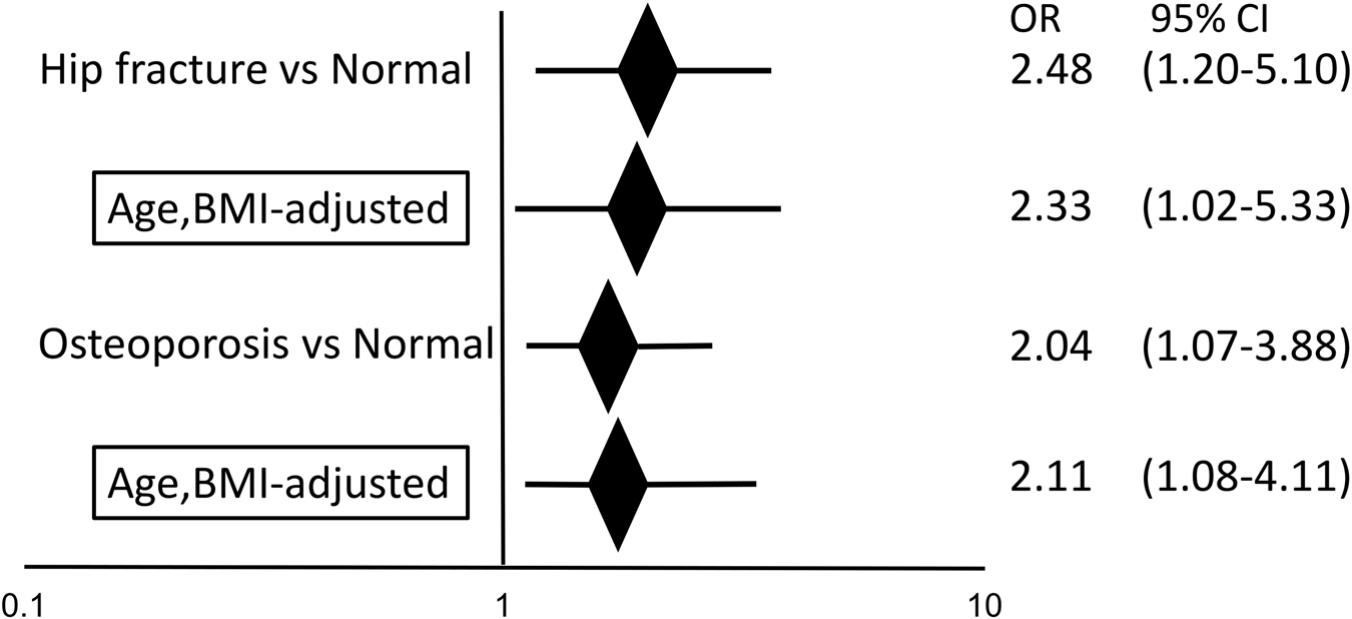
*ALDH2 rs671* is associated with hip fracture and osteoporosis. Odds ratio (OR) of *rs671* between hip fracture and normal groups, or between osteoporosis and normal groups was determined after adjustment for age and BMI.

Age, weight and BMI are parameters associated with osteoporosis development and hip fracture^11, 34, 35^. However, we found that the *rs671* remained associated with development of either hip fracture or osteoporosis, even after adjustment for age; body weight; BMI; age and body weight; or age and BMI (Table 3 and Fig. 4). These results suggest that *rs671* may represent a causative risk for hip fractures and osteoporosis.

**Table 3:** *ALDH2* rs671 genotype frequencies and odds ratios (OR) after adjustments.

	Crude OR	Age-adjusted OR	BW-adjusted OR	BMI-adjusted OR	Age and BW-adjusted OR	Age and BMI-adjusted OR
Hip fracture vs Normal	2.48 (1.20–5.10)	2.06 (0.93–4.58)	3.01 (1.36–6.63)	2.81 (1.32–6.01)	2.47 (1.07–5.71)	2.33 (1.02–5.33)
Osteoporosis vs Normal	2.04 (1.07–3.88)	1.95 (1.01–3.73)	2.21 (1.25–4.27)	2.21 (1.15–4.27)	2.34 (1.18–4.61)	2.11 (1.08–4.11)

### Osteoblast differentiation is inhibited by acetaldehyde and rescued by anti-oxidant treatment

**Figure 5:**
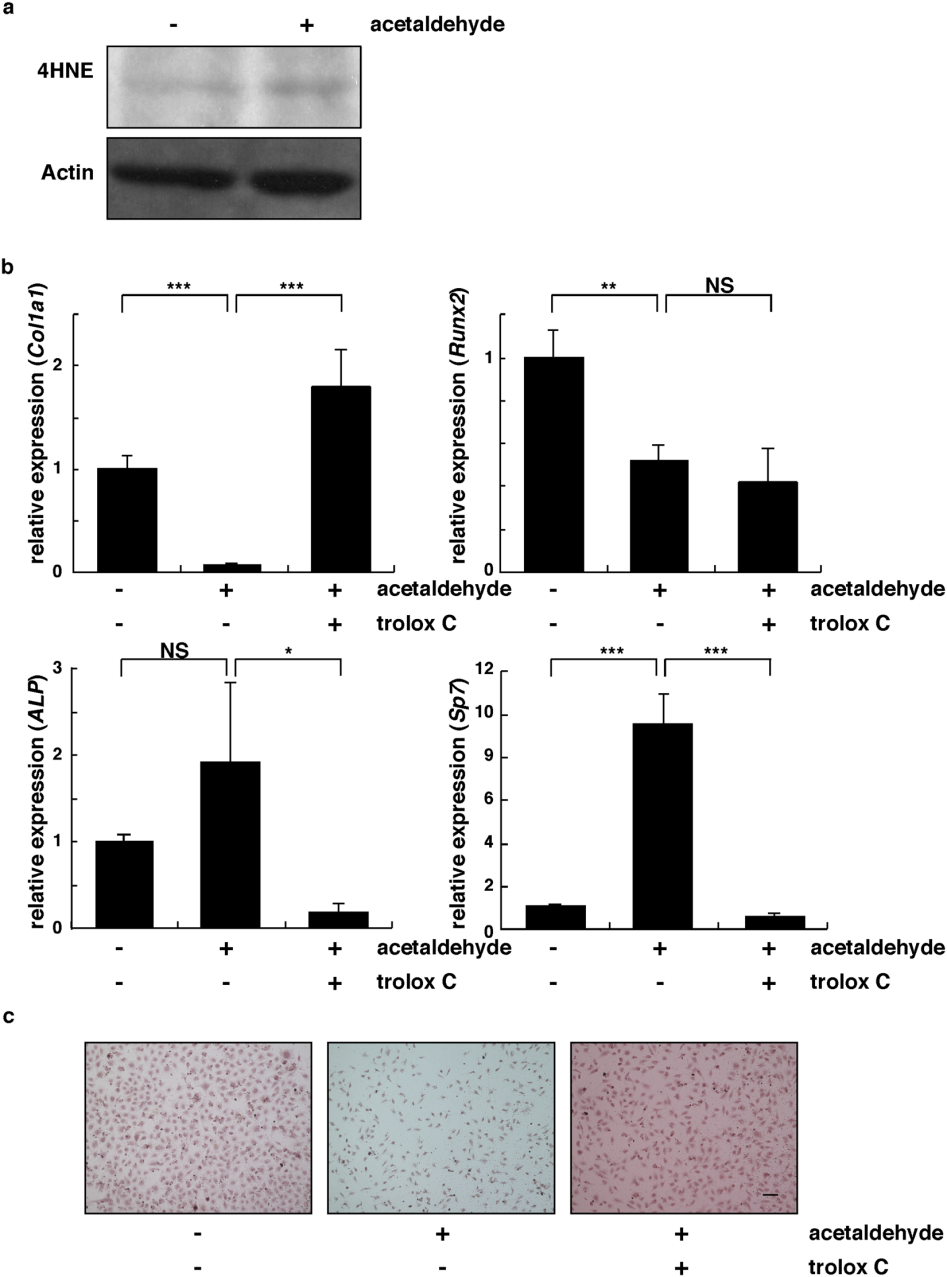
Osteoblast differentiation is inhibited by acetaldehyde and rescued by anti-oxidant treatment. (**a**) Osteoblastic MC3T3E1 cells were cultured in osteogenic medium with or without 0.04% acetaldehyde for 48 hours. Oxidative stress, as indicated by 4-hydroxynonenal (4HNE) levels, was then analyzed by western blot. Actin served as internal control. (**b**,**c**) MC3T3E1 cells were cultured in αMEM or osteogenic medium with or without 0.04% acetaldehyde and in the presence or absence of the anti-oxidant Trolox C (40 µM) for 48 hours (**b**) or seven days (**c**). Osteoblast differentiation was then analyzed by realtime PCR based on *Col1a1*, *Runx2*, *ALP* and *Sp7* expression (**b**) or alizarin red staining (**c**). Data represent mean *Col1a11*, *Runx2*, *ALP or Sp7* expression normalized to that of *β-actin* ± SD (*n* = 3). ***P < 0.001. Representative data of two independent experiments is shown.

The presence of the *rs671* polymorphism is thought increase acetaldehyde concentrations in the bloodstream, which may interfere with osteoblastogenesis^23^. Indeed, serum acetaldehyde levels are reportedly significantly elevated in ALDH2*2 relative to control mice^23^. Thus, we evaluated the effects of acetaldehyde in osteoblastic MC3T3E1 cells (Fig. 5). The compound 4-hydroxynonenal (4HNE) is an aldehydic product of lipid peroxidation implicated in pathological changes associated with oxidative stress and is a key mediator of oxidative stress-induced cellular responses^36^. We found that 4HNE levels, as determined by western blotting, were elevated in acetaldehyde-treated MC3T3E1 cells (Fig. 5a). Thus, we cultured MC3T3E1 cells with or without acetaldehyde in the presence or absence of the anti-oxidant Trolox C and analyzed potential effects on osteoblast differentiation (Fig. 5b). Osteoblastogenesis, as indicated by upregulation of the markers *collagen*, *type I*, *alpha 1* (*Col1a1*) and *runt-related transcription factor 2* (*Runx2*), was significantly inhibited by acetaldehyde (Fig. 5b). Acetaldehyde-dependent inhibition of *Col1a1* but not *Runx2* was rescued by Trolox C co-administration (Fig. 5b). However, expression of other osteoblastic markers, such as *osterix* (*Sp7*) and *alkaline phosphatase* (*ALP*), was upregulated by acetaldehyde and downregulated by Trolox C (Fig. 5b). Although expression of osteoblastic markers in MC3T3E1 cells in the presence of acetaldehyde and Trolox C varied, formation of alizarin red-positive mineralized nodules induced by osteogenic medium was blocked by acetaldehyde treatment, an outcome reversed by Trolox C treatment (Fig. 5c). These results suggest that overall osteoblastogenesis is inhibited by acetaldehyde, and that inhibition can be reversed by Trolox C.

### Osteoclast differentiation is inhibited by acetaldehyde and cannot be rescued by anti-oxidant treatment

**Figure 6:**
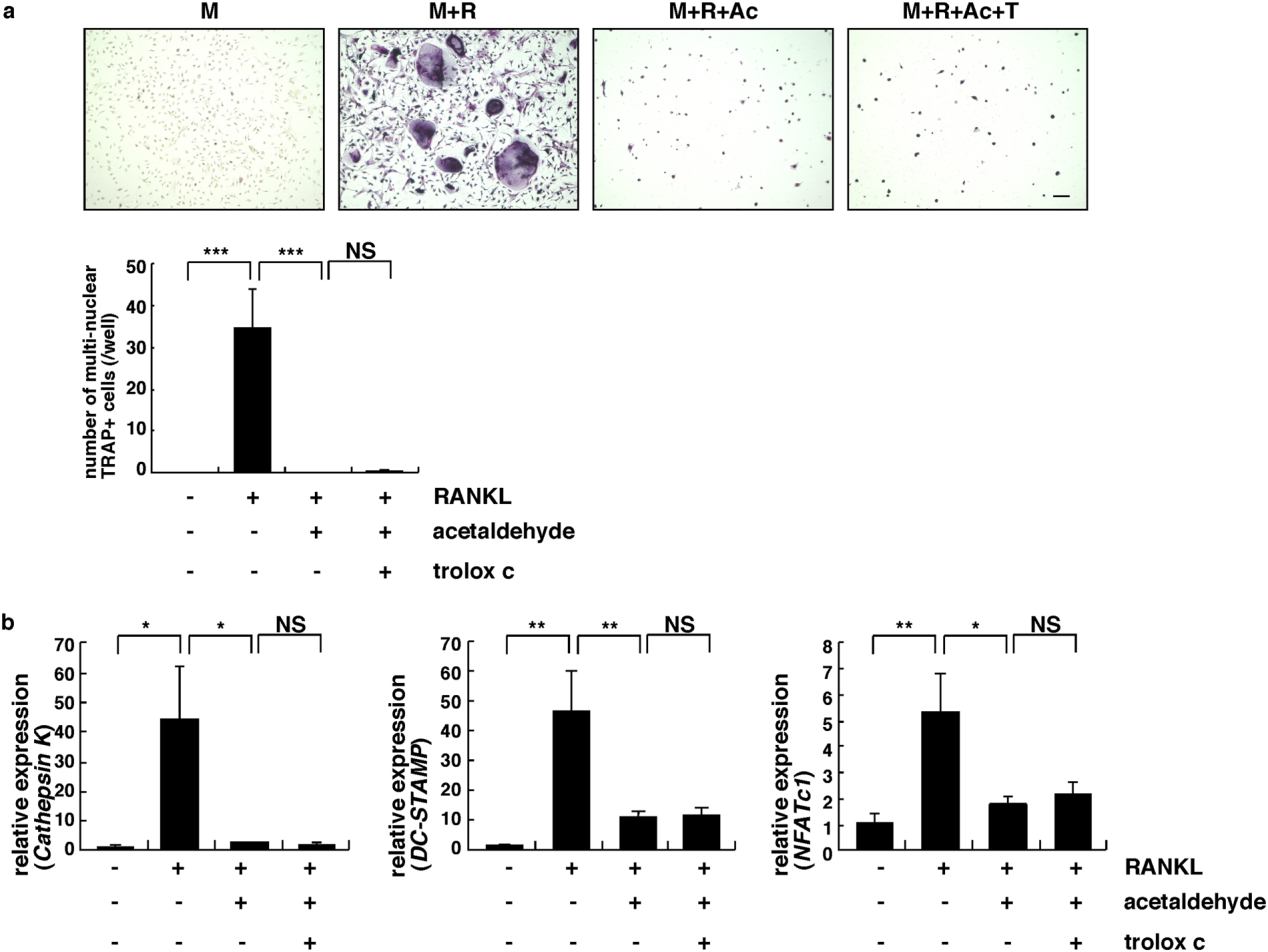
Osteoclast differentiation is inhibited by acetaldehyde and cannot be rescued by anti-oxidant treatment. (**a**,**b**) Wild-type mouse bone marrow-derived osteoclast progenitors were cultured in the presence of M-CSF (50 ng/ml) or M-CSF (25 ng/ml) plus RANKL (25 ng/ml) with or without 0.04% acetaldehyde and in the presence or absence of the anti-oxidant Trolox C (40 µM) for 5 days. Cells were then subjected to TRAP staining (**a**) or realtime PCR (**b**). Multi-nuclear TRAP-positive cells containing more than five nuclei were scored as osteoclasts (**a**). Data represent mean number of multi-nuclear TRAP-positive cells/well ± SD (*n* = 5). Realtime PCR data represent mean *Cathepsin K*, *NFATc1 or DC-STAMP* expression relative to *β-actin* ± SD (*n* = 3). ***P < 0.001. Representative data of two independent experiments is shown.

Finally, we asked the effects of acetaldehyde on osteoclast differentiation (Fig. 6). Osteoclastogenesis induced by treatment of mouse bone marrow-derived osteoclast progenitors with macrophage colony stimulating factor (M-CSF) and receptor activator of nuclear factor kappa B ligand (RANKL) was significantly inhibited by acetaldehyde (Fig. 6a). Osteoclast differentiation, as evaluated by multi-nuclear tartrate resistance acid phosphatase (TRAP)-positive cell formation and expression of the osteoclastic markers *Cathepsin K*, *nuclear factor of activated T cells 1* (*NFATc1*) and *dendritic cell specific transmembrane protein* (*DC-STAMP*), was significantly inhibited by acetaldehyde, but that inhibition was not reversed by Trolox C treatment (Fig. 6b).

## Discussion

Hip fracture is one of the most typical and severe bone fragile fractures among osteoporotic injuries. Hip fractures disturb activities of daily living and are even associated with mortality^37, 38^. Hip fracture history is significantly associated with increased probability of additional fracture^39^. Thus, preventing the first fracture is crucial. Risk factors such as aging, osteoporosis diagnosed as either hip/vertebral fracture or low BMD, muscle weakness and dementia are reportedly associated with hip fractures^35, 40–43^. A family history of hip fracture is also criteria for drug therapy to treat osteoporosis patients, and “parental hip fracture” is asked in calculating future hip fracture risks by FRAX^TM^. Several SNPs are reportedly associated with hip fracture^44^. Here, we show that *ALDH2 rs671* is a genetic factor associated with hip fractures, and that fracture risks more than double in its presence.

Interventions and treatments for osteoporosis can prevent hip fractures, and bisphosphonates, such as alendronate and risedronate, or the neutralizing RANKL antibody denosumab reportedly block hip fractures relative to placebo controls^45–47^. Osteoporosis is, however, not the only cause of hip fracture, and other risks, which cannot be determined by BMD, such as risk of falls or poor bone quality, remain even after osteoporosis treatment. *rs671* reportedly promotes low BMD in mice^23^ and is associated with human diseases such as Alzheimer’s disease and esophageal cancer^24, 26^. Thus, *rs671* may promote hip fracture via several mechanisms.

Tools available to predict fractures include FRAX^TM^, which can predict the probability of hip fractures within in the next decade. Hip fracture risk can be calculated by FRAX^TM^ in the absence of BMD analysis by considering age, gender, BMI, alcohol consumption exceeding three units per day and parental history of hip fracture. However, treatment history for osteoporosis and other conditions associated with hip fracture, such as diabetes, are not included in the FRAX^TM^ system. Significantly, *rs671* can be detected by genetic analysis and is strongly associated with skin flushing upon drinking alcohol. We found that the sensitivity and specificity to determine *rs671* by skin flushing upon drinking alcohol were approximately 80.0% and 92.3%, respectively, in our research subjects. Thus, *rs671* can be predictable by such responses to alcohol.

ALDH2*2 mouse models exhibit osteoblastic dysfunction due to oxidative stress mediated by accumulated acetaldehyde levels in the absence of alcohol intake^23^. Consumption of more than three units of alcohol daily is reportedly associated with hip fracture^9^, and ALDH2 functions in alcohol metabolism by clearing acetaldehyde^48^. Thus, alcohol consumption is considered a confounding factor for *rs671* in promoting hip fractures. However, no subjects in this study reported excessive alcohol consumption.

Osteoblastic apoptosis induced by acetaldehyde is reportedly effectively blocked by anti-oxidant treatment^23^. Here, we showed that osteoblastic differentiation was also inhibited by acetaldehyde and rescued in cultured cells by treatment with the anti-oxidant Trolox C. Similar to outcomes seen in osteoblasts, osteoclast differentiation was also significantly inhibited by acetaldehyde; however, that inhibition was not rescued by Trolox C. Since ALDH2*2 mice reportedly exhibit lower bone mass than controls^23^, inhibition of osteoblast function is considered dominant over changes in osteoclast formation in controlling bone mass under high acetaldehyde conditions. Further studies are needed, but hip fracture risk as predicted by *rs671* or excessive alcohol consumption may be prevented, at least in part, by administration of anti-oxidants without gene therapies for *rs671*.

## Conclusion

We identified *rs671* as potentially underlying, at least in part, a family history of hip fractures. The presence of *rs671* increases hip fracture risk more than two-fold compared with non-*rs671* carriers. The presence of *rs671* can be predicted by responses to alcohol such as skin flushing. Those risks may possibly be reduced, in part, by an administration of anti-oxidant without genetic correction of *rs671*. Finally, combined evaluation of *rs671*, BMD and FRAX^TM^ may provide a reliable index to predict hip fractures.

## Methods

### Subjects

This study was approved by an ethics committee at Keio University School of Medicine and carried out in accordance with guidelines approved by that committee. Informed consent was taken from all subjects prior to the study. All subjects underwent clinical and radiological examination by expert orthopedic surgeons. Subjects included 427 healthy postmenopausal Japanese women from five hospitals including our university hospital. Subjects with medical complications and/or who were under medical treatment known to alter bone metabolism were excluded from this study. The osteoporosis group (n = 248) was defined according to Japanese diagnostic criteria for primary osteoporosis^49^. The hip fracture group (n = 92), which was diagnosed with osteoporosis in the presence of hip fracture, was included in the osteoporosis group. Normal patients (n = 48) were defined by a T score greater than −2.5 without fragility fractures.

### Bone mass measurement

Lumbar spine BMD (anterior-posterior; L2-L4) and femoral neck BMD was measured by dual-energy X-ray absorptiometry (Lunar Prodigy DXA scanner; GE Medical Systems, Madison, Wisconsin, USA) and expressed in grams per square centimeter. Participant positioning and scan analysis procedures were standardized for all scans with CV < 0.01.

BMD was not measured in the hip fracture group (n = 92), a subgroup of the osteoporosis group, because the presence of hip fragility fracture met primary osteoporosis diagnostic criteria.

### Polymorphism analysis

Polymorphisms were analyzed by direct sequencing. Briefly, PCR was performed using ExTaq DNA polymerase (Takara Co., Kyoto, Japan) with 5′-TCCTGGGAGTGTAACCCATAAC-3′ (forward) and 5′-CCCTGAAGTCTCTCCCTCTTCT-3′ (reverse) primers. Amplified products were separated on a 1% agarose gels and purified using QIAquick gel extraction kits (Qiagen, Hilden, Germany). After purification, sequencing was performed using the BigDye Terminator (Applied Biosystems, Foster City, CA) with the forward primer, and sequence was analyzed using the ABI310 system (Thermo Fischer Scientific, Waltham, MA). ALDH2* heterozygotes and homozygotes were included in the mutation+ group.

### Osteoblast culture

Osteoblastic MC3T3E1 cells were cultured in alpha minimum essential medium containing 10% fetal bovine serum (αMEM) or in differentiation basal medium-osteogenic (osteogenic medium, Lonza, Tokyo, Japan) with or without 0.04% acetaldehyde (Wako Co. Ltd, Chuo-ku, Osaka, Japan) in the presence or absence of 40 µM Trolox C (Wako Co. Ltd) for 48 hours for realtime PCR and western blot or for seven days for alizarin red staining. RNA was extracted as described^23^, and osteoblastic differentiation was analyzed by realtime PCR for *Col1a1*, *Runx2*, *ALP or Sp7*/*β-actin* using SYBR Premix ExTaq II reagent and a DICE Thermal cycler (Takara Bio Inc., Kusatsu, Shiga, Japan) using the primers below. Results are shown as means ± SD.

*Col1a1*-forward: 5′-CATGTTCAGCTTTGTGGACCTC-3′

*Col1*-reverse: 5′-CCTTAGGCCATTGTGTATGCAG-3′

*Runx2*-forward: 5′-GACGTGCCCAGGCGTATTTC-3′

*Runx2*-reverse: 5′-AAGGTGGCTGGGTAGTGCATTC-3′

*ALP*-forward: 5′-CACCATTTTTAGTACTGGCCATCG-3′

*ALP*-reverse: 5′-GCTACATTGGTGTTGAGCTTTTGG-3′

*Sp7*-forward: 5′-ATGGCGTCCTCTCTGCTTGAG-3′

*Sp7*-reverse: 5′-CTTTCCCCAGGGTTGTTGAGTC-3′

*β-actin*-forward: 5′-TGAGAGGGAAATCGTGCGTGAC-3′

*β-actin*-reverse: 5′-AAGAAGGAAGGCTGGAAAAGAG-3′

For western blot analysis, whole cell lysates were prepared from cultured cells in RIPA buffer (1% Tween 20, 0.1% SDS, 150 mM NaCl, 10 mM Tris-HCl (pH 7.4), 0.25 mM phenylmethylsulfonylfluoride, 10 μg/mL aprotinin, 10 μg/mL leupeptin, 1 mM Na3VO4, and 5 mM NaF (Sigma-Aldrich Co.)) as described^50, 51^. Proteins were separated on SDS-PAGE, transferred to a PVDF membrane (EMD Millipore Corp.), and detected using anti-4HNE (Abcam) or anti-Actin (Sigma**-**Aldrich Co., St Louis, MO) antibodies.

For alizarin red staining, cultured cells were stained with 1% alizarin red, washed twice with distilled water and observed under a microscope.

### Osteoclast culture

Osteoclast differentiation was assessed as described^50^. Briefly, bone marrow cells were isolated from C57BL/6 wild-type mice and cultured 72 h in MEM (Sigma-Aldrich Co.) containing 10% FBS (JRH Biosciences) and GlutaMax (Invitrogen Corp.) with M-CSF (50 ng/mL, Kyowa Hakko Kirin Co.). Then, M-CSF-dependent adherent cells were harvested and cultured in 96-well plates (1 × 10^5^ cells per well) with M-CSF (50 ng/mL) and RANKL (25 ng/mL, PeproTech Ltd.) with or without 0.04% acetaldehyde and in the presence or absence of 40 µM Trolox C for five days. Cells were then subjected to TRAP staining and realtime PCR using the primers below.

*Ctsk*-forward: 5′-ACGGAGGCATTGACTCTGAAGATG-3′

*Ctsk*-reverse: 5′-GGAAGCACCAACGAGAGGAGAAAT-3′

*NFATc1*-forward: 5′-CAAGTCTCACCACAGGGCTCACTA-3′

*NFATc1*-reverse: 5′-GCGTGAGAGGTTCATTCTCCAAGT-3′

*DC-STAMP*-forward: 5′-TCCTCCATGAACAAACAGTTCCAA-3′

*DC-STAMP*-reverse: 5′-AGACGTGGTTTAGGAATGCAGCTC-3′

### Statistical analysis

Differences in age, height, body weight and BMI between hip fracture versus normal and osteoporosis versus normal groups were tested using a Mann-Whitney U-test. Logistic regression analysis was performed in the presence or absence of osteoporosis or hip fracture considered as a dependent variable, and gene polymorphism status, age, height, body weight, body mass index (BMI) as independent variables. The strength of an association between the polymorphism and hip fracture or osteoporosis was indicated by odds ratios (OR) and their 95% confidence intervals (CI). Data was also analyzed by the Chi-square test. An unpaired Student’s t-test was used to test differences in osteoblast differentiation, and significance was set at 0.05. All statistical analyses were performed with EZR version 3.2.2 (Saitama Medical Center, Jichi Medical University, Saitama, Japan)^52^.
